# Are Moral Emotions Key to Informed Risk Decisions? A Commentary on Sabine Roeser, Risk, Technology, and Moral Emotions

**DOI:** 10.1007/s11948-020-00193-6

**Published:** 2020-02-17

**Authors:** Madeleine Hayenhjelm

**Affiliations:** Department of Historical, Philosophical and Religious Studies, Umeå University, Umeå, Sweden

## Introduction

Are moral emotions key to moral knowledge? Are such emotions key to understanding the value of lay participation in risk decisions? Sabine Roeser’s book *Risk, Technology, and Moral Emotion* is about moral emotions, lay participation, and the role that both emotions and lay inclusion could and ought to play in risk decisions and risk communication.

The view that laypeople ought to be included in risk communication and risk decisions would be familiar to all readers of psychologist and decision-making analyst, Slovic (2000), and others. What Roeser does is question some of the presumptions in these arguments—first, the rationale that lay people ought to be included on the grounds that values are subjective, and second, the psychological view that emotions are irrational. Instead, Roeser argues, emotions are rational, cognitive, truth-apt, and serve as moral intuitions and thus give us access to moral knowledge. Indeed, moral emotions are necessary for moral knowledge, she argues. On these grounds, emotions ought to be included in moral decisions about risks, but checked by other higher-order, or second-order, emotions (such as compassion) and by reason. Equipped with a proper understanding of the role of emotions as trackers of moral values and moral salience, one can use this knowledge to dissolve stalemates in heated risk discussions. “Rather than ignoring emotions as in the technocratic approach, or taking emotions as endpoints of discussion as in the populist approach, or including the public only to the extent that it provides ‘rational arguments’ as in conventional participatory approaches,” Roeser suggests, “emotions should be the starting point of risk debates” (217). Essentially, moral emotions can point to underlying values that ought to form part of moral decisions about risk. The book picks up from where her previous book, *Moral Emotions and Intuitions*, left off (2011). It further develops and applies some of the core ideas developed there to risk policy and the lay-expert divide in risk communication.

Two themes run through the book: the first on moral emotions as moral knowledge and the second on democratic risk decisions inclusive of the voices of the lay public. The book is divided into three parts. In the first part of the book, she situates her own theory against the backdrop of previous theory on risk perception, psychological theories on decision-making processes, and ethical intuitionism. She argues that a more cognitive notion of emotions would challenge both the traditional ways of understanding the value of lay input into risk decisions and transcend the supposed dichotomy between mere “gut feelings” and rational decision-making processes. The second part presents her own theory of moral risk emotions, introducing her key concepts “risk emotions” and “moral risk emotions” as well as her idea that ethical intuitions are moral intuitions. The third part suggests ways to include moral emotions and lay emotions in risk decisions. This commentary will primarily focus on the middle part, which develops her own core ideas on moral emotions as necessary for moral knowledge.

The key idea of the book is that moral emotions form an essential moral insight, akin to moral intuitions, into moral truth. How convincing is the idea that moral emotions are necessary for moral knowledge? This commentary divides into the following parts: (a) an introduction to the central concept of “moral emotion” and the idea that moral emotions are necessary for moral knowledge, (b) an interpretative and critical reading of the moral emotions argument, (c) a critical discussion with three concerns raised, and (d) a brief return to the question of lay participation in light of the previous discussion.

## Moral Emotions

The core concept of the book is that of “moral emotions”—emotions that can serve as moral navigators to guide moral decisions. But what are these “moral emotions?” Roeser writes the following:

Moral emotions understood as felt value-judgments can play the role that intuitions and basic moral beliefs play for traditional intuitionists. Moral emotions are not deductive, inferential or strictly argumentative. Rather, through emotions we judge the moral value of a situation in a direct, experiential way. Moral emotions such as sympathy, compassion, shame and guilt provide us with access to the moral value of a situation, action or person. Moral emotions are fundamental moral experiences on which we can base further moral reasoning (2018, 92).

There are at least four central, recurring, and partly overlapping ideas in the book about the nature of moral emotions. These are:

1. Moral emotions are *cognitive* (but also conative and affective at the same time); they are felt value judgments (and if justified and true constitute moral knowledge) and have belief content.
2. Moral emotions are *analogous to sense perception*. Emotions can give us *direct perception* of morally salient features and values, and so can be regarded as analogous with sense perception.
3. Moral emotions are *intuitions.* They are non-inferential basic beliefs, akin to mathematical axioms, that can sustain further reasoning.
4. Moral emotions *track moral salience* or morally salient features in the world.

All these ideas are overlapping and support the general idea of moral emotions as central for moral knowledge. The strength of Roeser’s argument thus depends on how convincing each of these claims are and how consistent these claims are with one another. The claim that moral emotions are analogous to sense perception is not a separate idea in Roeser’s account from the idea that moral emotions are intuitions. However, while strongly linked, they are at least conceptually distinct enough to be worth listing as separate claims. In what follows, it is presumed that moral emotions are cognitive; there is a whole literature around that position and this is probably the least controversial of Roeser’s claims, but the other three claims will be addressed and discussed.

### Moral Emotions are Analogous to Sense Perception

In what way can moral emotions be analogous to sense perception? Both give us a direct experience of sorts. Furthermore, such experiences serve as a source of (fallible) knowledge. Roeser’s analogy claim thus goes beyond an analogy in terms of immediate impressions; there is also an analogy in how such impressions form the basis for knowledge. Roeser compares how one gains visual knowledge of color to how one gains moral knowledge from moral emotions:

1. Our *capacity* to feel moral emotions enables us to have
2. a “*felt value judgment*”—that is, we feel a moral emotion, which
3. paradigmatically (unless refuted) also comprises a moral *belief*
4. and *if* that moral belief *is justified and true* (plus possible additional conditions),
5. then we have emotional *knowledge* (90ff.).

This is analogous to how visual impressions form the basis for visual knowledge:

1. Our *capacity* to see colors *enables* us to have
2. color *vision*—that is, we see color, which
3. paradigmatically (unless refuted) comprises a color *belief*
4. and *if* that color belief is *justified and true* (plus possibly additional conditions),
5. then we have (visual) color *knowledge* (90).

Moral emotions thus form the basis for some kind of moral knowledge. This does not yet establish the second claim, that moral emotions also are necessary for moral knowledge, but merely establishes them as a possible source for such knowledge. Thus far, the idea is that emotions serve *as basic beliefs* that one can epistemically build upon.

### Moral Emotions are Intuitions and Basic Beliefs

The next step establishes how moral emotions can provide us with moral knowledge. Roeser contrasts the views of rationalists—that morality is objective and real, but emotions are subjective—with the view of sentimentalists that both morality and emotions are subjective (83). The rationalist position provides room for objective moral knowledge but not for any obvious epistemic role for moral emotions. The sentimentalist position provides an epistemic role for emotions but not for objective moral knowledge. Roeser’s own position is that moral realism is true, that emotions are objective and rational, and that emotions can convey moral knowledge in that they are able to track morally relevant features of the world. Should these morally salient features not be available without moral emotions, then they would also be necessary for moral knowledge. This is how Roeser spells out the central argument (84f):

P1: Reason is objective.

P2: Emotions are objective: emotions are necessary for moral knowledge.

P3: Ethics is objective.

Conclusion: Ethics is based on emotions [weak claim: epistemological, strong claim: ontological] (84).

This conclusion is, according to Roeser, consistent with two versions: a weaker epistemic claim and a stronger ontological claim. Roeser settles for the weaker claim:

Conclusion [weak]: Ethical *insight* is based on emotion, with emotions understood as a (fallible) source of moral knowledge (85).

To this, she adds three independently argued claims that together make up the core lines of argument for her theory (85):

Claim 1: Moral emotions are necessary for moral knowledge.

Claim 2: Moral emotions contain moral judgments.

Claim 3: Moral intuitions are paradigmatically moral emotions, with both understood as doxastic states.

Her main position, as expressed in the weak Conclusion above, is developed and defended against possible objections, in a step-by-step manner via the three Claims. According to Roeser, “the claims get stronger as they ascend from 1 to 3” (85). This is far from obvious as they stand. However, it seems that what increases from 1 to 3 is the richness of the account of moral emotions. Claim 1 introduces a minimal first step of the account: without moral emotions our moral judgment is found wanting. Claim 2 rejects the view of emotions as mere gut feelings and presents them as cognitive and on a par with sense perception as a source of knowledge. Claim 3 combines these two ideas with traditional intuitionism in ethical theory, but argues against the standard version where such intuitions are rational rather than emotional. Such ethical intuitions can either come in the form of basic beliefs, more or less akin to direct insight into moral axioms, or as holistic judgments over complex situations. Essentially, all three claims jointly support the core idea that moral emotions are necessary for moral knowledge understood in an objective way but they do so in varying degrees of theoretical richness. Central to the idea that moral emotions are necessary for moral knowledge (Claim 1) is what one could refer to as *the Damasio argument* based on the research conducted by Damasio (1994) on amygdala patients. Roeser writes:

But work from neuroscience (Damasio’s patients) and psychopathology (sociopaths) supports my claim that moral emotions are necessary for moral knowledge. Amygdala patients and sociopaths do often have intact or even highly functional rationality, but they lack emotions. Studies of these people show that they also lack the capacity to make concrete moral judgments. Indeed, Damasio’s amygdala patients are capable of making general moral judgements but not particular moral judgments, which requires taking into account concrete circumstances and contextual features (87ff).

This argument recurs a number of times in the book (and also in her previous book). In short: people who had damage to their amygdala were no longer able to *feel emotions* but they retained all their general knowledge and maintained the same level of IQ. These individuals changed after the damage and were no longer able *to act morally, make moral decisions, or interact morally*. They had “lost their capacity to make concrete moral and practical judgments” and “their personality had completely changed” (84). “They turned into rude people who do not know how to behave properly towards others and who cannot make practical and moral decisions in concrete situations” (ibid.). Hence, concludes Roeser, without moral emotions, one cannot have moral knowledge.

## A Reading

One of the core ideas of the book is the idea that moral emotions are necessary for moral knowledge. What this means and why this is a reasonable stance according to Roeser develops more or less organically through the book and often in response to the various literatures she engages with. This is the idea expressed in the Conclusion to her central argument and it is the position that develops across her three Claims. However, if the three Claims goes from thinner to richer in a more descriptive sense, then the reader is left without a straightforward argument for the combined “rich” position encompassing all three claims: that moral emotions are necessasry for knowledge (Claim 1) because they track moral salience and values akin to sense perception (Claim 2) and that moral intuitions are moral emotions (Claim 3), in essence, a form of direct but fallible perception into moral truth. What speaks for this rich conclusion? One reading of Roeser’s main argument of the book would thus be as follows:

1. [*The Cognitive Claim*] Emotions are doxastic states and (to some significant degree) also rational (in the sense that they are cognitive and sensitive to reason).
2. [*The Moral Salience Claim*] Emotions track moral salience or morally salient properties. (They “read” the morally relevant aspects of the world, so to speak.)
3. [*The Basic Belief Claim*] Emotions are basic beliefs and perceive moral reality. (They track moral reality as their default, even if fallible.)

Hence: To the extent that *only* moral emotions can “read” the world morally and *only emotions* can track moral reality, emotions are necessary for moral knowledge (and if not, they may still track morality in some or most cases).

In order to assess the strength of these claims, more needs to be said about how to understand moral knowledge.

### Three Kinds of Moral Knowledge

How plausible the above position is depends on how we understand moral knowledge. Roeser herself does not define moral knowledge. There are at least three very different readings one could make of the term: (1) moral knowledge as the practical know-how of how to relate to others and know what to do in particular contexts, (2) moral knowledge as an analytical skill to understand and analyze complex moral situations, and (3) moral knowledge as a hypothetical collection of all that is true about morality. The first would be a skill and capacity to “read” and relate to the world morally as a moral agent. The second would be an intellectual capacity to understand and analyze situations morally. The third would be a bulk of knowledge that can be written down, communicated, explained, and passed on. This would suggest three very different claims about moral emotions being necessary for moral knowledge.

1. Moral emotions are necessary in order to “read” a concrete situation and view it from a moral point of view and to be motivated to act in response.
2. Moral emotions are necessary in order to understand moral facts (when reading about them, or comparing situations, etc.) and to be able to understand complex moral situations.
3. Moral emotions are necessary for there to be anything called moral knowledge that could be written down in a book, taught to others, and so on.

The Damasio cases obviously support the claim that moral emotions are necessary at least for the first type of knowledge and possibly also for the second. It is less clear to what degree they can also be taken to support moral knowledge in the third sense, when abstracted away from the immediate situation. Roeser’s claim that moral emotions are necessary for moral knowledge, however, goes beyond moral know-how and definitely includes truth-apt propositions about morality. The moral knowledge she has in mind must thus include moral facts of some kind. However, it is not very clear what these facts consist of. Moral emotions are mostly described as trackers of moral values and moral salience; however, even if it is granted that that values and salience could be described as facts with propositional content (“x is valuable,” “x is morally salient,” and so on) it is less obvious that such propositions make up the bulk of moral knowledge. Thus, the question remains how to get from those kinds of evaluative facts to more deontic facts and facts about the right thing to do. In other words, even if it is granted that moral values and moral salience are facts and that moral emotions are necessary in all of the three ways mentioned above, this still does not settle the question about to what degree such knowledge makes up the bulk of moral knowledge. Three possible answers come to mind. First, this could mean that moral emotions yield direct perceptions of values and moral salience that such facts constitute the entirety of objective moral knowledge. Perhaps it could be argued that the right thing to do is determined by whatever is rational to do in light of both descriptive and evaluative facts. In short, the view here is that all moral facts consist of evaluative facts and based upon such facts we can make morally informed decisions., beyond that, overall judgments about what to do are about deliberation and judgment but not about objective facts. Secondly, it could mean that moral facts constitute the “raw data” from which moral knowledge is constructed. It could be argued that any moral principle that determines rightness of actions presumes some knowledge about values and moral salience, but that the bulk of moral knowledge is constituted by knowledge at the level of principles and their application. Moral knowledge would then consist in general principles and the conclusions derived from them. We would never be able to formulate such principles without the original emotional moral experiences, but once we have knowledge of the principles these can largely replace the need for moral emotions to guide us to right and wrong. Moral emotions would be necessary for such principles in a formative stage (but not beyond that). Finally, it could mean that moral emotions also provide knowledge of moral rightness as moral facts, perhaps as an intuitive judgment about what the right thing to do is after reflection and deliberation or as a direct response to a complex situation. Moral emotions, on this view, would both serve as a way to access basic moral facts about values and as a moral arbitrator for right and wrong in more holistic assessments. In the first case, the immediate felt value judgments gained via moral emotions, checked by reason and other emotions such as compassion, constitutes the bulk of moral knowledge. In the second case, there is a bulk of general objective moral knowledge. Such knowledge presumes moral emotions at the initial stage but these play a lesser role in the whole of moral knowledge. In the third case, moral emotions are both necessary in the sense of providing evaluative knowledge of values and moral salience and necessary in order to know what the right thing to do is (and thus replaces the need for principles). Moral knowledge could also include other kinds of (mid-level) moral reasoning, but both the initial seeds and the conclusions would depend on moral emotions. Roeser largely objects to “master principles” and defends particularism as an important part of her framework. This rules out the second position. The first position is compatible what Roeser writes, and with ethical intuitions as basic beliefs, but seems unnecessarily restrictive. But once we expand the notion of moral knowledge beyond the immediate case, it is hard to see how we can avoid generalised conclusions and principles, thus restricting emotions, beyond the initial tracking of moral salience and values, to the role of guidance in how and when to apply them. The third position could possibly be compatible with ethical intuitions understood as holistic judgments of complex situations that Roeser mentions but does not fully explore (93f).

## A Strong Claim and Three Concerns

How far can moral emotions and moral perceptions of moral salience and values take us towards knowledge about what the right thing to do is? In the strongest possible case, moral emotions would be a source of moral knowledge over abstract moral and complex situations as much as over immediate and concrete ones; they would be a source of objective truths about duties and rightness as much as they would be of values and moral salience, and they would get the normative content right by default and only get things wrong if there was some identifiable source of distortion or some similar external force at work. This could be framed in the following way:

*The Strong Claim:* Moral emotions are a source of objective moral knowledge and moral truth in both abstract and concrete moral situations, they track objective values and deontic properties, and, even if fallible, they tend to track moral truth as their default.

If the strong claim holds, this would provide a very strong case for moral emotions as a significant contributor to moral knowledge. Here the focus will be on three critical concerns that such a position would be vulnerable to. The first one is about the scope: how well can moral emotions provide moral knowledge in cases that extend beyond immediate and concrete moral situations? The second one is about objectivity: how is the difference between accurate moral emotions and moral emotions that merely reflect pre-existing beliefs and experiences to be distinguished? The third one is about accuracy: is it plausible to assume that moral emotions tend to get things right by default? These partially overlap but they will be discussed in turn.

### From Concrete to Abstract and Complex Situations

First, let us begin with the most clear-cut and convincing cases in favor of Roeser’s account: the most immediate and simple moral situations. Imagine a baby being abandoned outdoors in the baking sun; an elderly woman not able to cross the street on her own; a pick-pocket removing a smartphone from someone’s back pocket; a car speeding through a quiet street full of children; soldiers firing into a group of peaceful protesters, and so on. The moral facts are directly observable to the observing moral agent in much the same way that a snake is observable to the person who has a visual impression of it. Furthermore, all the relevant moral details of the case seem to be, more or less, limited to what can be so perceived. The question is whether the color vision analogy only holds when the moral situation is observable in this way or whether moral emotions are equally reliable when it comes to more distant or more abstract moral situations. The above type of immediate situations could, for instance, be contrasted with distant moral situations where the whole moral situation can only be grasped in the abstract. Risk decisions are seldom about such immediate moral situations. In the simple cases, the perception analogy seems convincing. But in what sense are moral emotions a reliable, but fallible, guide when it comes to moral situations that are not immediate? For instance, in cases when what is before the moral agent is only a small fragment of a complex moral whole, where any immediate conclusions may be false since they are only part of the picture? Roeser also suggests that intuitions about risks can only be reliable when it comes to the moral aspects but not the “quantitative” aspects (45) and that the latter must be supplied by science. However, the worry here is not about completeness of information, but about the nature of moral emotions as a source of knowledge.

It seems that what moral emotions primarily can do is to pick out what is morally salient about a particular case in a sense that is relevant to that particular case. This suggests three hypotheses: (a) moral emotions can only track moral truth in immediate and concrete cases; (b) moral judgments based on moral emotions can be transferred, by means of analogy or similar, to abstract but discrete cases, and (c) moral emotions can track moral truth also in contemplated moral situations that are not immediate or concrete. If the first, then moral emotions would only be relevant to a very limited number of moral cases and not much relevant at all to most cases of risk decision given that risks per definition are about what has not yet occurred and cannot yet be perceived. Furthermore, it may not be known who the potential victims of a risk are, how it could affect them, when or where the risk could materialize. The effects of a risk can be far-reaching (affecting future generations as in the case of climate change), distant (affecting distant others), diffuse (as in “invisible threats” such as air pollution), affect anonymous others or be generally unpredictable if new. The second position would allow for moral conclusions or moral observations in one case to apply also to other types of cases, either via some kind of general moral principle or by means of analogy. Roeser seems favorable to both (a) and (b). She writes: “Most intuitionists emphasize a bottom-up approach to moral knowledge: we initially make particular moral judgments, based on which we can form more general moral judgments” (92). However, the two do not seem to be epistemically on a par. She writes that “moral emotions are especially well suited to particular moral judgments” (93) and furthermore that “moral emotions in dilemmatic or complex situations are more fallible” (120). Given the very strong claims about moral intuitions and about their context dependency, it is far from clear how much of the epistemic role of moral emotions can be transferred to the complex case. The third position would allow for moral emotions to apply equally to abstract moral situations as much as concrete ones. However, even if the moral emotions were to be the same, the reliability does not seem to be the same, given that moral emotions could only react to the situation as presented and thus how an abstract case is described would already determine what is morally salient in some sense.

### Moral Emotions are Objective

The underlying idea behind the idea that emotions are objective is that emotions track real features of the world. It is thus the objects of emotions that are real and in that sense objective. A feeling of fear may indicate that something truly is dangerous, as in the case of fear when observing a snake. Emotions can rightfully pick out real objects as objects for concern. However, moral emotions do not seem to be objective in the way that we can assume that two individuals would judge the same situation in the same way or even regard the same moral features as the most salient. Rather, how they would assess any given situation may reflect their moral beliefs and previous experiences more than the actual situation. For instance: what one person views as an act of cruelty another may view as an act of justice. This could be explained by different moral beliefs and it could be explained by different moral experiences. In the case of the snake, there is an objective state of the world that would settle the matter: a snake observed either is or is not of a poisonous kind. In the case of moral emotions, there may be an objective truth to the matter; something either is or is not morally wrong, but such objectivity would lie in moral truth not in observable facts. Roeser argues that moral emotions need to be checked by other emotions and by reason. But without some kind of moral criterion of rightness it is not clear how to determine when such “checks” gets things right and when not. In short, it seems that moral emotions are both subjective and objective in the ways described above.

### Moral Emotions Track Moral Truth

Moral emotions are moral intuitions in Roeser’s account. Moral emotions are fallible, just like other kinds of knowing, but on the whole they tend to get things right. But how plausible is this? If moral emotions were anything like the capacity to see colors, then they would, on the whole, get things right, and when they do not, there are simple explanations that count as distortions in one way or another. What makes such claims about color visions plausible is that the distortion tends to have a source outside the capacity itself. When something is perceived to be one thing but it turns out to be something else, this can in most cases be verified by the same capacity of vision with the distorting element removed, as when a distorted perception is due to faulty lighting or a strange angle. But if moral judgments are informed by beliefs, then correcting one moral judgment would be to replace it with one based on a different belief. Roeser also agrees that moral emotions need to be checked via reason, as well as by universal emotions. But it is hard to see how this would work if there is no objective standard for when something is right or wrong—and without general normative principles and moral intuitions as the ultimate source of moral knowledge, it is hard to see where we would find this. Roeser appeals to “compassion” but even if compassion were to be the right tool, it is hard to see how we could know that it would be the right tool by her reasoning.

## Democratic Decisions and Moral Emotions

Let us now turn to the second question: To what extent can lay input provide necessary moral knowledge? The theoretical core chapters of the book point in the direction of an intuitionist argument for lay inclusion: the input of laypeople are necessary because moral emotions are necessary for moral decisions about risks. This would be a very interesting claim if successful. But how could that be the case? As a reason for lay participation, it is a weak claim. In fact, both democratic arguments and arguments from moral contractualism would give stronger cases for lay inclusion, since democratic legitimacy could never be achieved without the democratic inclusion of those affected and moral rightness could never be achieved without agreement between those affected (Scanlon 2000). By contrast, it is only if lay individuals’ input is necessary for moral emotions to be given due consideration that such participation would be necessary on the grounds of moral emotions. But it is very hard to see that moral emotions could never be considered without lay participation.

Even though much empirical risk perception research suggests that laypeople differ to experts in their understanding of risk and that they include more ethical considerations, this, by itself, does not imply that experts could not take this broader view. A single, very competent, moral agent with the right kind of emotional and rational capacities could be sufficient to provide the necessary moral input. Thus, the need to include lay populations would be conditional on experts not nurturing their moral emotions or not consulting an ideal emotional and rational adviser. A significant part of Roeser’s overall framework is a commitment to the irreducible pluralism of moral aspects of situations and she would most likely object to the single ideal knower having privileged access to moral truth. Such an idea could point towards the epistemic superiority of a multitude or a broad set of “knowers,” but does not by itself give a compelling argument for lay inclusions on such grounds.

In fact, Roeser ends up arguing for a much weaker claim about lay participation, one more to the effect that laypeople’s moral emotions are rational and could add valuable knowledge to risk decisions. Roeser addresses what she refers to as the “lay puzzle” (103ff). On the one hand, it is acknowledged that laypeople view risks differently than do experts, as demonstrated by the risk perception studies by Slovic and colleagues. On the other hand, it is also acknowledged that laypeople’s risk perception is at least partially based on emotions. And, on the assumption that emotions are unreliable as a source of information to base risk decisions on, a contradiction arises in that laypeople’s contributions both are and are not normatively valid input. The lay puzzle can be solved, Roeser argues, if the assumption that emotions are unreliable as a source of information (because they are subjective) is replaced with the claim she has already argued for: that moral emotions are a valuable source of objective moral knowledge. Here, however, the earlier claims about moral emotions seem somewhat watered down to much weaker claims: “Taking emotions as the starting point of risk debates may reveal genuine ethical concerns that should be taken seriously” (133). She adds, “It might also show biases and irrational emotions that can be addressed by information that is presented in an emotionally accessible way. Purely rational reflection would not be able to provide us with the imaginary power that we need to envisage future scenarios, take part in other people’s perspectives and evaluate their destinies” (ibid.). Here moral emotions are depicted less as necessary to moral knowledge and more as one source of valuable moral input to moral decisions and as a tool in communication.

On this weaker view, the idea is not so much to justify lay participation with an appeal to moral emotions, as to add another aspect to the existing participatory framework: there are already strong democratic and possibly also epistemic reasons to include lay populations in decisions about risks, and their emotional input, something that is often held against such lay participation, is, in fact, an additional reason for it. The reasoning here seems more pragmatic: lay populations are often dismissed for being emotional or included on the basis of a subjective view of morality,  but they can be included without subscribing to a subjective view and need not be dismissed on an objective view. If emotions are morally informative in an objective sense, then there is no reason to exclude lay populations on the grounds that they are emotional. This seems right and important on its own, but does not fully explain how to transfer the stronger claims about moral emotions as intuitions to the context of risk decisions and lay participation. It seems that moral emotions are simultaneously given at least two different epistemic statuses: as direct but fallible perceptions of moral truth and as more opaque pointers towards underlying values that are indispensable contributions that require additional scrutiny. Both ideas are interesting, but it is not clear how they can be both. This brings us back to an earlier point. Roeser's account of moral emotions is the most convincing when defending emotions as trackers of moral salience and moral values in concrete moral situations where what can be observed and what can be “felt” largely overlap. The case for moral emotions as necessary for moral knowledge in a more general and theoretical sense seems much weaker. The former claim is important and interesting and does seem to suggest a necessary role for moral emotions with regards to moral knowledge in this limited sense.
